# Different spatial patterns of brain atrophy and global functional connectivity impairments in major depressive disorder

**DOI:** 10.1007/s11682-016-9645-z

**Published:** 2016-10-20

**Authors:** Chuanjun Zhuo, Jiajia Zhu, Chunli Wang, Hongru Qu, Xiaolei Ma, Wen Qin

**Affiliations:** 1 Department of Radiology and Tianjin Key Laboratory of Functional Imaging, Tianjin Medical University General Hospital, No. 154, Anshan Road, Heping District, Tianjin, 300052 China; 2 Department of Psychiatry Functional Neuroimaging Laboratory, Tianjin Mental Health Center, Tianjin Anding Hospital, Tianjin, China; 3 Department of Psychiatry, Wenzhou seventh people’s hospital, Wenzhou, Zhejiang China; 4 Institute of Mental Health, Jining Medical University, Jining, Shandong China

**Keywords:** Major depressive disorder, Grey matter volume, Functional connectivity density, Magnetic resonance imaging, Resting state

## Abstract

Alterations in grey matter structure and functional connectivity (FC) are pathological features of major depressive disorder (MDD). However, the relationship between brain structural and functional impairments in MDD remains uncertain. Thus, the aim of this study was to investigate the relationship between alterations in grey matter volume (GMV) and resting-state global functional connectivity density (rs-gFCD) in MDD. Forty-five patients with MDD and 48 healthy controls underwent structural and resting-state functional magnetic resonance imaging (fMRI). GMV and rs-gFCD maps of each subject were created, and the intergroup differences in these neuroimaging metrics were evaluated. Compared to healthy controls, patients with MDD displayed reduced GMV in the left insula, putamen, amygdala and hippocampus (mainly in the limbic system). In contrast, decreased rs-gFCD was observed in the left postcentral and precentral gyri, right fusiform gyrus and lingual gyrus (mainly in the sensory system). Furthermore, we found no significant correlations between GMV and rs-gFCD in these brain regions. Finally, overlap ratio analysis demonstrated that the spatial distribution patterns of intergroup differences on the GMV and rs-gFCD maps were distinct. Our findings suggest that the presence of distinct spatial distribution patterns of brain atrophy and global FC impairments might be pathological features of MDD.

**Electronic supplementary material:**

The online version of this article (doi:10.1007/s11682-016-9645-z) contains supplementary material, which is available to authorized users.

## Introduction

Major depressive disorder (MDD) is typically characterized by pervasive and persistent sadness and feelings of helplessness and worthlessness (American Psychiatric Association 2013; Kahn 1975). Over the past decade, this disease has become the second leading cause of disability worldwide (Brundtland 2001; Rosenblat et al. 2015). In the last ten years, due to advances in neuroimaging techniques, many studies using magnetic resonance imaging (MRI) have explored the pathological features of MDD in terms of both structural and functional alterations in the brain. These studies have provided many key findings that have enhanced our understanding of the neurobiology of depression (Northoff et al. 2011; Phillips et al. 2015; Savitz and Drevets 2009; Wise et al. 2014).

Grey matter volume (GMV) and functional connectivity (FC) can be used in clinical applications as biomarkers of neuropsychiatric disorders (Ashburner and Friston 2000). Aberrant GMV has been reported in brain regions of the limbic system in patients with MDD, such as the temporal lobe, basal ganglia, amygdala, hippocampus, insular cortex and orbitofrontal cortex (Lorenzetti et al. 2009; Peng et al. 2011; Stratmann et al. 2014; Zou et al. 2010). In addition, aberrant FC has been reported in brain regions related to the processing of emotional, cognitive, sensorimotor and visual information in MDD, such as in the prefrontal lobe, anterior cingulate cortex, pre-central gyrus, post-central gyrus, fusiform and lingual gyrus, amygdala, and thalamus, etc. (Iwabuchi et al. 2015; Mulders et al. 2015; Peng et al. 2015; van Tol et al. 2013; Zhang et al. 2015). The aforementioned studies suggested that structural and functional alterations coexisted in patients with MDD. Because brain structure and functional organization are internally interactive, it is important to investigate the relationship between the changes in brain structure and function in MDD. However, to the best of our knowledge, few studies have investigated the relationships between the spatial distribution patterns of GMV and FC alterations.

Voxel-based morphometry (VBM) is an unbiased and data-driven method to quantify tissue content in the brain (Ashburner and Friston 2000). This algorithm can segment brain tissue into grey matter, white matter and cerebral blood flow components and then register these components in a common template to compare them voxel by voxel. VBM has been widely used to identify regional variability in brain GMV in both healthy subjects and patients with neuropsychiatric disorders (Peng et al. 2011; Wang et al. 2014). In contrast, functional connectivity density mapping (FCDM) is a voxel-wise, data-driven method that has been commonly used to examine the density distribution of whole-brain resting-state FC (Tomasi and Volkow 2010, 2011b, 2011c), i.e., resting-state global functional connectivity density (rs-gFCD). rs-gFCD has also been referred to as the degree of centrality (Buckner et al. 2009) or intrinsic connectivity contrast (Martuzzi et al. 2011), and brain regions with high rs-gFCD are considered hubs interconnecting distinct, functionally specialized systems. Recent studies showed that rs-gFCD could be used as a biomarker of neuropsychiatric disorders (Buckner et al. 2009; Tomasi et al. 2015).

In the current study, using VBM and FCDM techniques, we investigated the relationships between the spatial distribution patterns of GMV and rs-gFCD alterations in patients with MDD. Based on the reports of earlier studies, we hypothesized that patients with MDD shared similar spatial distributions between altered GMV and rs-gFCD in the limbic system, while they demonstrated unique spatial distributions of altered rs-gFCD in the sensory system. We also investigated whether the brain regions exhibiting altered GMV or rs-gFCD were correlated with clinical variables.

## Materials and methods

### Subjects

Patients were recruited from the inpatient and outpatient departments of Tianjin Mental Health Center and Tianjin Anning Hospital. Healthy controls were recruited from the local community via advertisements. A total of 93 right-handed subjects, including 45 patients with depression (19 men, mean age 38.8 ± 13.3 years) and 48 healthy controls (21 men, mean age 38.6 ± 10.5 years), participated in this study after providing written informed consent in accordance with the regulations of the Medical Research Ethics Committee of Tianjin Medical University. Diagnoses of depression were confirmed by two psychiatrists, using the Structured Clinical Interview for the DSM-IV (SCID) (First et al. 1997). The exclusion criteria included significant neurological or physical diseases, past head injury with loss of consciousness, history of drug or alcohol abuse or dependency, pregnancy or MRI findings of contraindications. Additional exclusion criteria for the healthy control subjects were a lifetime history of a psychiatric disorder or having a first-degree relative with a mood or anxiety disorder. Depression severity was rated using the Hamilton Depression Rating Scale (HDRS) (Fleck et al. 2004). Twenty-six patients were taking selective serotonin reuptake inhibitors (SSRIs) as an antidepressant medication (paroxetine, citalopram, or sertraline), eight patients were taking noradrenergic and specific serotonergic antidepressants (mirtazapine), four patients were taking a serotonin-noradrenaline reuptake inhibitor (SNRI) as an antidepressant medication (venlafaxine), one patient was taking a melatonin receptor agonist (agomelatine), two patients were taking Deanxit, and four patients were using Chinese herbal medicines. In addition, 8 patients were using mood stabilizers (valproate or atypical antipsychotics), 6 patients simultaneously used two types of antidepressants, and 10 patients used sedative-hypnotic drugs or anxiolytics (Table 1). The current medication duration varied from 7 days to 3 months, and only 2 patients were in complete remission during the scanning period.

**Table 1:** Demographic and clinical characteristics of the subjects enrolled in this study

Characteristics	MDD	HC	Statistics	*P* value
Number of subjects	45	48		
Age (years)	38.8 (13.3)	38.6 (10.5)	*t* = 0.062	0.951
Sex ratio (female/male)	26/19	27/21	χ^2^ = 0.022	0.882
HDRS score	27.8 (10.6)	N/A		
Illness duration (months)	36.5 (62.0)	N/A		
Concomitant use of psychopharmacologic drugs		N/A		
Antidepressants (%)	41/45			
Chinese herbal medicines (%)	4/45			
Combined with mood stabilizers (%)	8/45			
Combined with two antidepressants (%)	6/45			
Combined with hypnotic-sedative drugs or anxiolytics (%)	10/45			

Data are shown as means (SD)

*HC* healthy control subjects, *HDRS* Hamilton Depression Rating Scale, *MDD* major depression disorder patients

### MRI data acquisition

MRI data were acquired using a 3.0 T MR system (Discovery MR750, General Electric, Milwaukee, WI, USA). Tight but comfortable foam padding was used to minimize head motion, and earplugs were used to reduce scanner noise. Sagittal 3D T1-weighted images were acquired using a brain volume sequence with the following parameters: repetition time (TR) = 8.2 ms; echo time (TE) = 3.2 ms; inversion time (TI) = 450 ms; flip angle (FA) = 12°; field of view (FOV) = 256 mm × 256 mm; matrix =256 × 256; slice thickness = 1 mm, no gap; and 188 sagittal slices. Resting-state functional MRI (fMRI) data were acquired using a gradient-echo single-shot echo planar imaging sequence with the following parameters: TR/TE = 2000/45 ms; FOV = 220 mm × 220 mm; matrix =64 × 64; FA = 90°; slice thickness = 4 mm; gap =0.5 mm; 32 interleaved transverse slices; and 180 volumes. All of the subjects were instructed to keep their eyes closed, relax, move as little as possible, think of nothing in particular, and not fall asleep during the fMRI scans.

### fMRI data preprocessing

Resting-state fMRI data were preprocessed using SPM8 software (http://www.fil.ion.ucl.ac.uk/spm). For each subject, the first 10 volumes were discarded to allow the signal to attain equilibrium, while the participants adapted to the scanning noise. The remaining volumes were corrected for the acquisition time delay between slices. Realignment was then performed to correct the motion between time points. The fMRI data for all of the subjects were within the defined motion thresholds (i.e., translational and rotational motion of less than 2 mm and 2°, respectively). We also calculated the frame-wise displacement (FD), which indexes the volume-to-volume changes in head position. There was no significant difference in the mean FD (*t* = −1.286; *P* = 0.202) between the patients (0.087 ± 0.040) and controls (0.099 ± 0.044). Several nuisance covariates (six motion parameters, their first-order derivatives, and the average BOLD signals of the ventricles and white matter) were regressed out of the data. Recently, global signal regression has been considered a controversial topic in resting-state fMRI analyses (Fox et al. 2009; Murphy et al. 2009) because global signals have also been found to reflect neurobiologically important information (Scholvinck et al. 2010). Thus, we did not remove the global signal in the fMRI data preprocessing. Recent studies have reported that the signal spike caused by head motion significantly contaminated the final resting-state fMRI results, even after regressing out the linear motion parameters (Power et al. 2012). Therefore, we further regressed out the spike volumes when the FD of the specific volume exceeded 0.5. The datasets were then band-pass filtered over a frequency range of 0.01 to 0.08 Hz. In the normalization step, individual structural images were linearly (12 affine parameters) co-registered with the mean functional image. The structural images were then segmented and normalized to the Montreal Neurological Institute (MNI) space using a high-level nonlinear warping algorithm, according to the diffeomorphic anatomical registration through exponentiated Lie algebra (DARTEL) technique (Ashburner 2007). Finally, each filtered functional volume was nonlinearly transformed in the MNI space using the deformation parameters of the two co-registration steps and was resampled into a 3-mm cubic voxel.

### GMV calculation

The GMV of each voxel was calculated using the VBM8 toolbox (http://dbm.neuro.uni-jena.de/vbm.html). Structural MR images were segmented into GM, white matter and cerebrospinal fluid using the standard segmentation model. After initial affine registration of the GM concentration map to the MNI space, GM concentration images were nonlinearly warped using the DARTEL technique (Ashburner 2007), and the results were resampled to a voxel size of 1.5 mm × 1.5 mm × 1.5 mm. The relative GMV of each voxel was obtained by multiplying the GM concentration map by the nonlinear determinants derived from the spatial normalization step. Finally, the GMV images were smoothed using a Gaussian kernel of 6 mm × 6 mm × 6 mm full width at half maximum (FWHM).

### Calculation of rs-gFCD

The rs-gFCD of each voxel was calculated using an in-house script written on a Linux platform, according to the method described by Tomasi and Volkow (2010, 2011b, 2011c). We first computed Pearson’s linear correlations between the time series of each pair of all of the GM voxels and obtained a whole-brain FC matrix for each subject. The computation was constrained within a cerebral GM mask, which was generated by thresholding (a threshold of 0.2) a prior GM probability map in SPM8. Pairs of voxels with a correlation coefficient of *r* > 0.6 were considered significantly connected. For a given voxel ×_0_, rs-gFCD was computed as the number of functional connections between ×_0_ and all of the other GM voxels that satisfied the correlation coefficient threshold (i.e., *r* > 0.6). To confirm whether the correlation threshold would influence intergroup comparisons, we further calculated the rs-gFCD using the same process, except for a correlation coefficient threshold of *r* > 0.4. To minimize variability across subjects, grand mean scaling of rs-gFCD was performed by dividing the rs-gFCD value of each voxel by the mean rs-gFCD value of all of the cerebral GM voxels. Finally, the rs-gFCD maps were spatially smoothed using a 6 mm × 6 mm × 6 mm FWHM Gaussian kernel.

### Calculation of resting-state functional connectivity (rs-FC)

To investigate the FC that contributed to the intergroup differences in rs-gFCD, brain regions with significant group differences in rs-gFCD were defined as seed regions. Then, rs-FC analysis was performed separately for each seed region. For each subject, rs-FC was defined as the Pearson’s correlation coefficient between the mean time course of the seed region and that of each voxel within the cerebral GM. Then, Fisher’s r-to-z transformation was performed on the rs-FC maps to improve the normality of the data. Finally, the rs-FC maps were smoothed using a Gaussian kernel of 6 mm × 6 mm × 6 mm FWHM.

### Statistical analysis

First, voxel-wise intergroup comparisons of GMV and rs-gFCD (with r > 0.6) were performed using a GLM, controlling for age and sex. Correction for multiple comparisons was performed using the cluster-level topological false-discovery rate (FDR) method in SPM8 (Chumbley et al. 2010), considering a corrected cluster-level threshold of *P* < 0.05 and a single-voxel threshold of *P* = 0.01. According to a recent study (Eklund et al. 2016), fMRI cluster-wise inferences disproportionately inflate false-positive rates if a cluster-defining threshold (CDT) of *P* = 0.01 is used. To validate the results, we repeated the voxel-wise intergroup comparisons of GMV and rs-gFCD using a permutation-based inference tool for nonparametric statistics (“randomize”, part of FSL). The number of permutations was set to 5000, and the significance threshold was set at *P* < 0.05 after correcting for family-wise error (FWE) using the threshold-free cluster enhancement (TFCE) option in FSL.

To validate whether the correlation threshold would influence inter-group comparisons, we further compared inter-group differences in rs-gFCD with a correlation coefficient of *r* > 0.4, and we performed inter-group analysis using the same GLM model. In addition, to exclude the possible effect of head size on rs-gFCD changes, we repeated the voxel-based rs-gFCD comparisons with the whole-brain GMV as an additional covariate.

To quantify the similarity between the spatial patterns of functional and structural alterations, we examined the overlap ratio (OR) of the brain regions displaying significant intergroup differences in GMV and rs-gFCD. In addition, to exclude the possibility that thresholding might influence the OR calculation (for example, a looser statistical threshold may cause larger OR, vise versa), a spatial correlation coefficient (SCC) between the T maps of GMV and rs-gFCD intergroup comparsions cross voxels was carried out using Pearson’s correlation analyses.

To test whether the alterations in GMV or rs-gFCD were correlated with the severity of depressive symptoms, we extracted the mean GMV or rs-gFCD values of each cluster as identified by the voxel-based GMV or rs-gFCD comparative analyses, and we performed region-of-interest (ROI)-based correlation analyses with the HDRS score. Because most of the variables were not normally distributed, Spearman’s correlation coefficients were used, and a significance threshold of *P* < 0.05 was set. To determine whether there was an association between GMV and rs-gFCD changes, we performed ROI-based Spearman’s correlation analyses between the rs-gFCD of each cluster exhibiting altered rs-gFCD and the GMV of each cluster exhibiting altered GMV in MDD patients.

For the rs-FC comparison, individuals FC maps were entered into a random-effects one-sample t-test in a voxel-wise manner using SPM8. FWE correction with *P* < 0.05 was used to identify the brain regions showing significant, positive correlations with each seed region. Then, a GLM-based two-sample t-test was performed within the positive rs-FC masks to compare the intergroup differences in rs-FC while controlling for age and sex. The performance of multiple comparisons was corrected using the cluster-level topological FDR method (*P* < 0.05).

## Results

### Demographic and clinical characteristics

The demographic and clinical characteristics of the participants are shown in Table 1. There were no significant intergroup differences in sex (χ^2^ = 0.022; *P* = 0.882) or age (*t* = 0.062; *P* = 0.951). The mean total HDRS score was 27.8 ± 10.6, and the mean duration of MDD was 36.5 ± 62.0 months.

### Intergroup differences in GMV and rs-gFCD

**Fig. 1:**
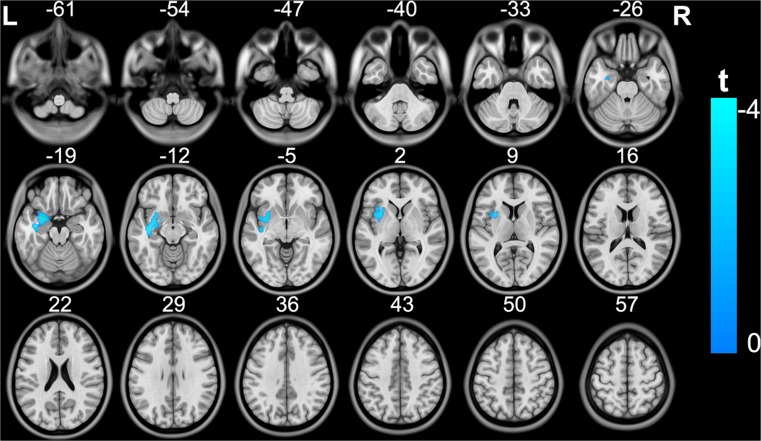
Brain regions exhibiting significant differences in GMV between patients with MDD and healthy subjects. The coloured bar represents the t value. GMV, grey matter volume; MDD, major depressive disorder

**Fig. 2:**
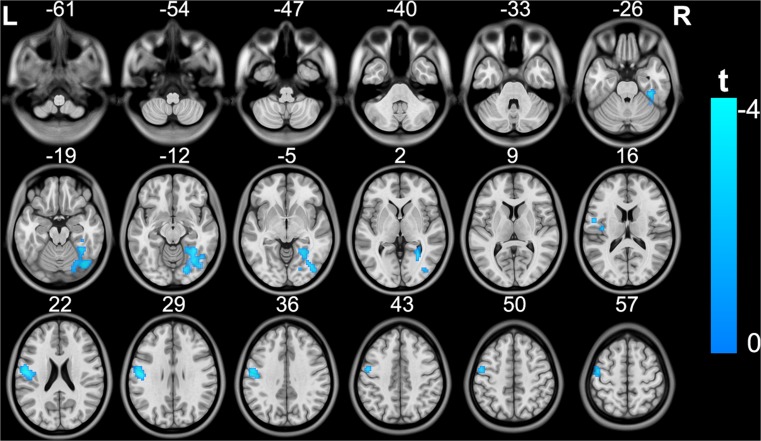
Brain regions exhibiting significant differences in rs-gFCD between patients with MDD and healthy subjects. The coloured bar represents the t value. rs-gFCD, resting-state global functional connectivity density; MDD, major depressive disorder

Compared with the control group, the MDD patient group exhibited significantly decreased GMV in the left insula, putamen, amygdala and hippocampus (cluster size =2270; peak MNI coordinates: x/y/z = −25.5/4.5/−21; peak *t* = −4.1), most of which are components of the limbic system (Fig. 1) (topological FDR q < 0.05; corrected cluster size 2270 voxels). MDD patients also showed significantly decreased rs-gFCD in the left postcentral and precentral gyri (cluster size =226; peak MNI coordinates: x/y/z = −54/−9/24; peak *t* = −4.2), which are ascribed to the sensorimotor system, and decreased rs-gFCD in the right fusiform and lingual gyri (cluster size =328; peak MNI coordinates: x/y/z = 27/−66/−18; peak *t* = −3.9), which are ascribed to the visual system (Fig. 2) (topological FDR q < 0.05; corrected cluster size 226 voxels).

For nonparametric statistics, the MDD patients showed significantly decreased rs-gFCD in the left sensorimotor cortex and bilateral visual cortex (Fig. S1) (*P* < 0.05, FWE corrected), which were similar to those in the parametric statistics. Despite no significant inter-group difference, we found a trend towards a significant reduction in GMV (Fig. S2) (*P* < 0.06, FWE corrected) in the left insula, putamen, amygdala and hippocampus, although the spatial extent was smaller than those in the parametric statistics.

The distributions of brain regions with decreased rs-gFCD at the threshold of *r* > 0.4 were similar to those observed at the threshold of *r* > 0.6 (Fig. S3). Furthermore, the aforementioned brain regions with altered rs-gFCD in MDD patients remained after correcting for whole-brain GMV (Fig. S4) (topological FDR q < 0.05; corrected cluster size of at least 215 voxels), indicating that the altered rs-gFCD in MDD patients could not be explained by individual variance in global GMV. We did not find increased GMV or rs-gFCD in any brain region of the MDD subjects.

**Fig. 3:**
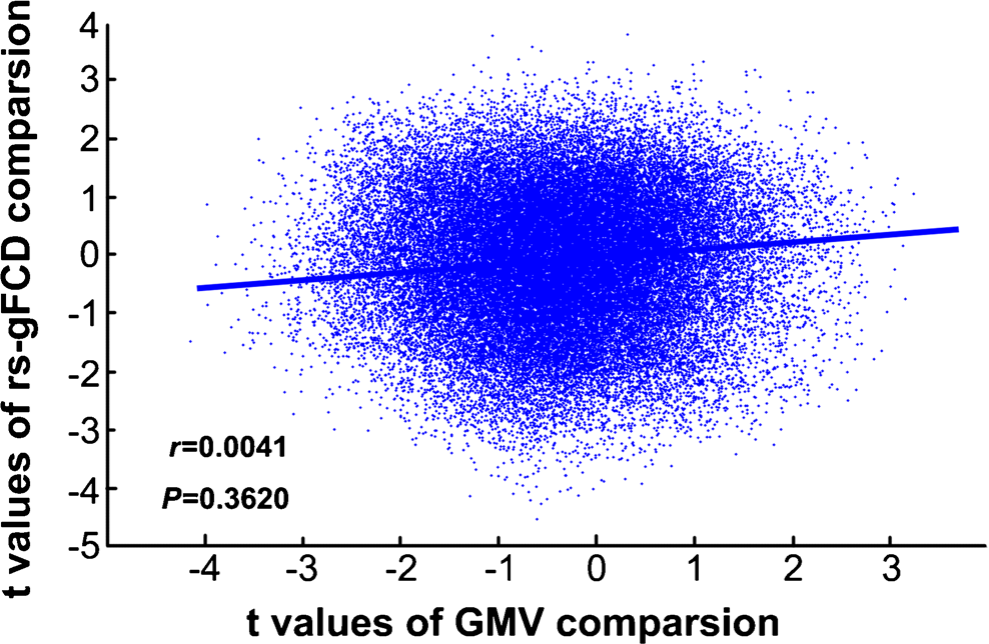
The spatial correlation coefficient (SCC) between the T maps of intergroup differences in GMV and rs-gFCD. rs-gFCD, resting-state global functional connectivity density; GMV, grey matter volume

Overlap ratio analysis demonstrated that there was no overlap (OR =0) between the spatial distributions of the intergroup differences in GMV and rs-gFCD, and Spatial correlation analysis also showed there was no statistical correlation (*r* = 0.004, *P* = 0.362) between the spatial distributions in T maps of GMV and rs-gFCD intergroup differences (Fig. 3), indicating that the spatial distribution patterns of decreased GMV and rs-gFCD were distinct in MDD patients.

### Intergroup differences in rs-FC

**Fig. 4:**
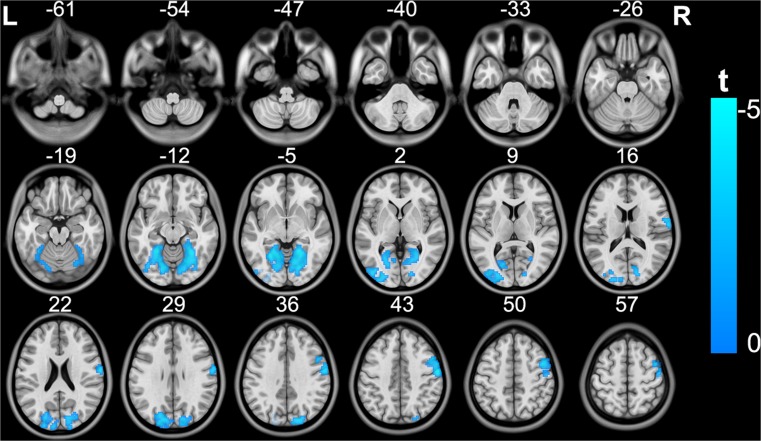
Brain regions exhibiting significant differences in rs-FC of the right visual region between patients with MDD and healthy subjects. The coloured bar represents the t value. rs-FC, resting-state functional connectivity; MDD, major depressive disorder; ROI, region of interest

MDD patients showed decreased FC between the right visual seed and the bilateral visual regions and right sensorimotor region, compared to the healthy subjects (Fig. 4) (topological FDR q < 0.05; corrected cluster size 273 voxels). However, there was no significant intergroup difference in the rs-FC of the left sensorimotor seed (topological FDR q < 0.05).

### Correlation between clinical symptoms and neuroimaging parameters

In MDD patients, we did not find any statistical correlation between the HDRS scores and the examined metrics (GMV of limbic regions, Spearman’s rho = −0.055, *P =* 0.721; rs-gFCD of sensorimotor regions, Spearman’s rho = −0.169, *P =* 0.267; rs-gFCD of visual regions, Spearman’s rho =0.180, *P =* 0.238). In addition, no significant correlation was identified between the decrease in GMV in the limbic regions and the decrease in rs-gFCD in either the sensorimotor regions (Spearman’s rho = −0.009; *P* = 0.953) or the visual regions (Spearman’s rho =0.064; *P* = 0.678) in MDD patients.

## Discussion

In this study, we found that both GMV and rs-gFCD were reduced in MDD patients compared to healthy controls. The reductions in GMV were primarily located in the limbic system (i.e., left insula, amygdala, and hippocampus). In contrast, the decreases in rs-gFCD were primarily located in brain regions participating in the sensory system (i.e., left postcentral and precentral gyri, right fusiform gyrus, and lingual gyrus). Furthermore, the spatial distribution exhibiting reduced GMV did not overlap or correlate with that exhibiting decreased rs-gFCD. These results indicated that the spatial distribution patterns of decreased GMV and rs-gFCD were distinct in MDD patients. Finally, no brain regions exhibited statistical correlations of either rs-gFCD or GMV with clinical symptoms.

In the present study, the brain regions with reduced GMV in patients with MDD were primarily located in the limbic system. This result was consistent with the early findings of many previous studies (Lorenzetti et al. 2009; Peng et al. 2011; Stratmann et al. 2014; Zou et al. 2010). We also observed decreased rs-gFCD in the sensorimotor and visual cortices in MDD patients. Furthermore, we found that MDD patients showed decreased FC between the right visual seed and the bilateral visual regions and the right sensorimotor region, as compared to the healthy subjects, which is consistent with the rs-gFCD findings in this study and provides additional information about the FC of the visual areas that were impaired in MDD. However, we did not find significant differences in FC between the left sensorimotor seed and other brain voxels, which might have been caused by the failure of seed-based FC analysis in detecting abundant sub-threshold changes. Our findings indicate that rs-gFCD analyses might be more sensitive in detecting the global FC changes than seed-based FC analysis in cases where many concurrent subthreshold FC changes are presented. These findings are consistent with several previous reports, such as decreases in the regional spontaneous neural activity of the pre- and postcentral gyri in MDD (Liang et al. 2013), decreases in the amplitude of low-frequency fluctuation (ALFF) in the right lingual gyrus (Wang et al. 2012), decreases in rs-FC of the calcarine cortex (Guo et al. 2013) and lingual gyri (Veer et al. 2010), and decreases in activation of the fusiform gyrus in response to positive stimuli in MDD patients (Stuhrmann et al. 2011). Recently, Northoff et al. proposed that the deficit in resting-state activity of the sensorimotor network might be an important biomarker that reflects the clinical psychomotor symptoms of MDD, such as psychomotor agitation or retardation (Northoff 2016a; Northoff 2016b; Vares et al. 2015). Our finding of decreased rs-gFCD in the sensorimotor system provides additional evidence for this newly developed hypothesis. Previous studies have suggested that the visual system plays a key role in the perception of emotions during the presentation of facial stimuli (Dichter et al. 2009; Tao et al. 2013) and that functional deficits in the visual processing system can increase sensitivity to depression-related stimuli (sad words, sad faces) (Surguladze et al. 2004; Weniger et al. 2004). Our finding of decreased rs-gFCD in the visual system also supports the hypothesis of a deficit in visual network functioning in MDD patients. Although most of the exiting studies of rs-FC in MDD did not identify abnormalities of FC in the visual cortex (Mulders et al. 2015), the most recent study reported abnormal FCD in the visual cortex in first-episode, drug-naive adult patients with MDD (Zou et al. 2016), consistent with our findings. The reason for this difference might be that most previous studies used either seed-based correlation or independent component analysis (ICA), while our study employed FCD analysis, which characterizes rs-FC from the perspective of graph theory. Thus, these methods might have different sensitivity and specificity in detecting abnormal FC in MDD.

It should be noted that we did not detect aberrant FC in brain regions related to emotional and cognitive processing, such as the frontopariental network, attentional network, default mode network, emotional-related network, etc. (Kaiser et al. 2015; Perrin et al. 2012; Wang et al. 2015). This finding was also inconsistent with a recent study showing decreased rs-gFCD in the MCC and increased rs-gFCD in the dorsal visual areas (Zhang et al. 2015). According to previous studies, several factors, such as age, sex, genetics, therapeutic agents, acquisition parameters, fluctuations of conscious states and the selection of different preprocessing strategies, contributed to individual variability in FCD (Liao et al. 2013; Tomasi and Volkow 2010, 2011a, 2011d). We postulated that differences between the involved subjects might have accounted for the inconsistencies between our findings and previous findings (Zhang et al. 2015). For example, our study included 26 female and 19 male MDD patients, whereas Zhang’s study included 9 women and 12 men, creating the opposite sex ratio; moreover, racial differences might also explain the differences between the findings of our study and those of previous studies (for example, all Chinese in the present study while German in Zhang et al). In addition, several studies have reported that disease state and illness duration could influence spontaneous neural activity in the brains of MDD patients to varying degrees (Guo et al. 2012; Wang et al. 2013). In our study, the MDD patient group included 7 patients experiencing first major depressive episodes, 2 patients were in complete remission, and 36 patients had a persistent depressive illness duration of more than 3 months. In contrast, all of the subjects in Zhang et al.‘s study were experiencing acute depressive episodes (Zhang et al. 2015). Moreover, the patients in the present study and Zhang et al.‘s study were all taking antidepressants, mood stabilizers or other therapeutic agents, and the differences in therapeutic agents between the two studies likely explained the aforementioned different findings to some extent. Finally, different FC-related techniques might have their own merits (and/or flaws) in depositing different aspects of rs-FC. For example, rs-gFCD is a powerful data-driven technique for quantifying the FCD of each brain voxel. This metric is also known as the degree of centrality (Buckner et al. 2009) or intrinsic connectivity contrast (Martuzzi et al. 2011), and brain regions with high rs-gFCD are considered functional hubs that are highly connected to the rest of the brain. This metric totals the contributions of all of the connected voxels with a certain voxel, thus having greater sensitivity in detecting concurrent global FC changes; however, it might also cover some changes when these connectivities show opposite directions (for example, some increase, while others decrease). This hypothesis was supported by a recent meta-analysis; for example, decreased rs-FC between the regions of the dorsal attention network (DAN) and frontopariental network (DMN) but increased rs-FC between the regions of the DAN and default mode network (DMN) were shown in MDD, which could theoretically weaken the detection of rs-gFCD alterations in the DAN (Kaiser et al. 2015). Because, since then, only 2 studies (including the present study) have explored the alterations in rs-gFCD in MDD patients, these findings should be validated in the future using larger sample sizes across different populations, illness durations, clinical symptoms and states, and so on.

Notably, in the present study, we aimed to investigate the spatial relationships between structural atrophy and functional disconnection in patients with MDD. The coexistence of structural and functional brain alterations may constitute the neural mechanism of MDD, and clarifying this issue could facilitate the identification of treatment targets for MDD. Moreover, structural alterations are likely to affect functional dynamics, yet persistent functional alterations can induce structural changes by affecting synaptic plasticity. Thus, we previously hypothesized that patients with MDD might share similar spatial distributions between altered GMV and rs-gFCD in some networks, such as the limbic system. Unexpectedly, in this study, the MDD-related GMV reduction did not spatially overlap with the MDD-related rs-gFCD decrease. The distinct spatial distribution between structure and function in MDD was also reported by Guo et al., who found that brain regions exhibiting reduced GMV did not overlap with those exhibiting altered ALFF (Guo et al. 2014a). Our findings suggest that depression might be associated with distinct patterns of brain structural and functional impairments, which are characterized by grey matter atrophy in the limbic system and a reduction in the global functional communication of the sensory-related circuits. However, because of the nature of rs-gFCD in characterizing global functional communication efficiency (unable to determine which functional connectivity is changed), we cannot exclude that the functional connectivity patterns of certain hubs of the limbic system are not influenced in MDD. In fact, early studies showed hypo-connectivity of the hippocampus, amygdala and insula in MDD (Kaiser et al. 2015).

We did not find any statistical correlations between the neuroimaging parameters (GMV or rs-gFCD) and clinical symptoms. According to previous studies, due to the poor reproducibility of results across different studies, the relationships between brain structural and functional alterations and the clinical variables associated with MDD remain controversial (Cao et al. 2012; Drevets et al. 2008; Dunlop and Mayberg 2014; Dutta et al. 2014; Gong and He 2015; Graham et al. 2013; Grieve et al. 2013; Guo et al. 2014b; Marchand et al. 2012; Mulders et al. 2015; Wang et al. 2013; Wolfers et al. 2015). Many confounding factors, such as differences in social and demographic characteristics within the patient population (e.g., age at first episode, positive or negative family history of psychological disorders, sample size, extent of depressive symptoms, current and chronic medication status, neuroimaging techniques, and MRI data processing methods), might underlie the failure to achieve a consensus on this matter (Dunlop and Mayberg 2014; Dutta et al. 2014; Gong and He 2015; Graham et al. 2013; Mulders et al. 2015; Wolfers et al. 2015). Because longitudinal follow-up studies could more comprehensively control for the aforementioned confounding factors and could dynamically characterize the development of the pathological features of MDD while providing accurate “state” and “trait” markers for early diagnosis or objective predictors for personalized treatment of MDD, we recommend large-sample longitudinal follow-up studies enrolling high risk cases and first-episode, drug-naive MDD patients and the use of uniform neuroimaging acquisition and analytical methods to acquire and analyse the neuroimaging and clinical data at different time points (such as the time points of the first episode, remission, and recurrence). Furthermore, follow-up analyses of the differences in neuroimaging data between different treatment outcomes in MDD patients (to identify non-treatment-resistant or treatment-resistant depression) should be included.

Some limitations of this study should be noted. First, the patients were taking antidepressants, mood stabilizers or other therapeutic agents, and it was previously reported that structural and functional alterations are likely influenced by these therapeutic agents (Chi et al. 2015; Dusi et al. 2015; Fang et al. 2015; Greicius et al. 2007). Unfortunately, this difficult question has not yet been satisfactorily resolved (Greicius et al. 2007; Guo et al. 2011) because the dosages of different antidepressants cannot be converted to a uniform equivalent in the same manner as for antipsychotics, which can be converted to a chlorpromazine equivalent. Therefore, this variable could not be used as a covariate to control for the effects of antidepressants. A longitudinal follow-up study enrolling only first-episode drug-naive MDD patients would be preferable in the future to control for these confounding factors. Second, although we found decreased rs-gFCD in the sensorimotor and visual regions of MDD patients, we did not evaluate the sensorimotor and visual functions of both MDD and NC subjects, which prevented us from drawing firm conclusions concerning the functional importance of rs-gFCD abnormalities. Third, many other analyses of both structural and functional organization, such as cortical thickness and surface area, anatomical connectivity, diffusion quantification, anatomical and functional network, etc., could be conducted simultaneously to comprehensively reveal the pathological features of MDD.

## Limitation

According to a recent study (Eklund et al. 2016), fMRI cluster-wise inferences have disproportionately inflated false-positive rates if a cluster-defining threshold (CDT) of *P* = 0.01 is used. We followed the Reviewer’s suggestion and performed the statistical analysis with CDT *P* = 0.001. Unfortunately, our original results could not pass the multiple comparison correction.

However, to validate our results, we repeated the voxel-wise intergroup comparisons of GMV and rs-gFCD using a permutation-based inference tool for nonparametric statistics (“randomize”, part of FSL). The number of permutations was set at 5000, and the significance threshold was set at *P* < 0.05 after correcting for family-wise error (FWE) using the threshold-free cluster enhancement (TFCE) option in FSL.

The results of nonparametric statistics are shown in Figs. R1 and R2 (please see Figs. S1 and S2 in the Supplementary Materials). MDD patients showed significantly decreased rs-gFCD in the left sensorimotor cortex and bilateral visual cortex (Fig. R1) (P < 0.05, FWE corrected), similar to those in the parametric statistics. Despite a lack of significant inter-group difference, we found a trend towards a significant reduction in GMV (Fig. R2) (*P* < 0.06, FWE corrected) in the left insula, putamen, amygdala and hippocampus, although the spatial extent was smaller than those in the parametric statistics.

## Conclusion

In summary, our study found that reduced GMV was primarily located in the limbic system, while decreased rs-gFCD was primarily located in the sensorimotor and visual systems, and the spatial distributions exhibiting reduced GMV and rs-gFCD did not overlap or correlate with each other. Our findings suggest that depression might be associated with distinct spatial patterns of brain grey matter and global functional communication impairments.

## Electronic supplementary material

Fig. S1 (DOCX 801 kb)

Fig. S2 (DOCX 788 kb)

Fig. S3 (DOCX 799 kb)

Fig. S4 (DOCX 802 kb)
