# Supplementary material for: Different spatial patterns of brain atrophy and global functional connectivity impairments in major depressive disorder
Source: Brain Imaging Behav. 2016 Oct 20;11(6):1678–89. doi: 10.1007/s11682-016-9645-z (PMC5707231; doi:10.1007/s11682-016-9645-z)
Supplement: Supplementary file 1 — (DOCX 801 kb) [file 11682_2016_9645_MOESM1_ESM.docx]

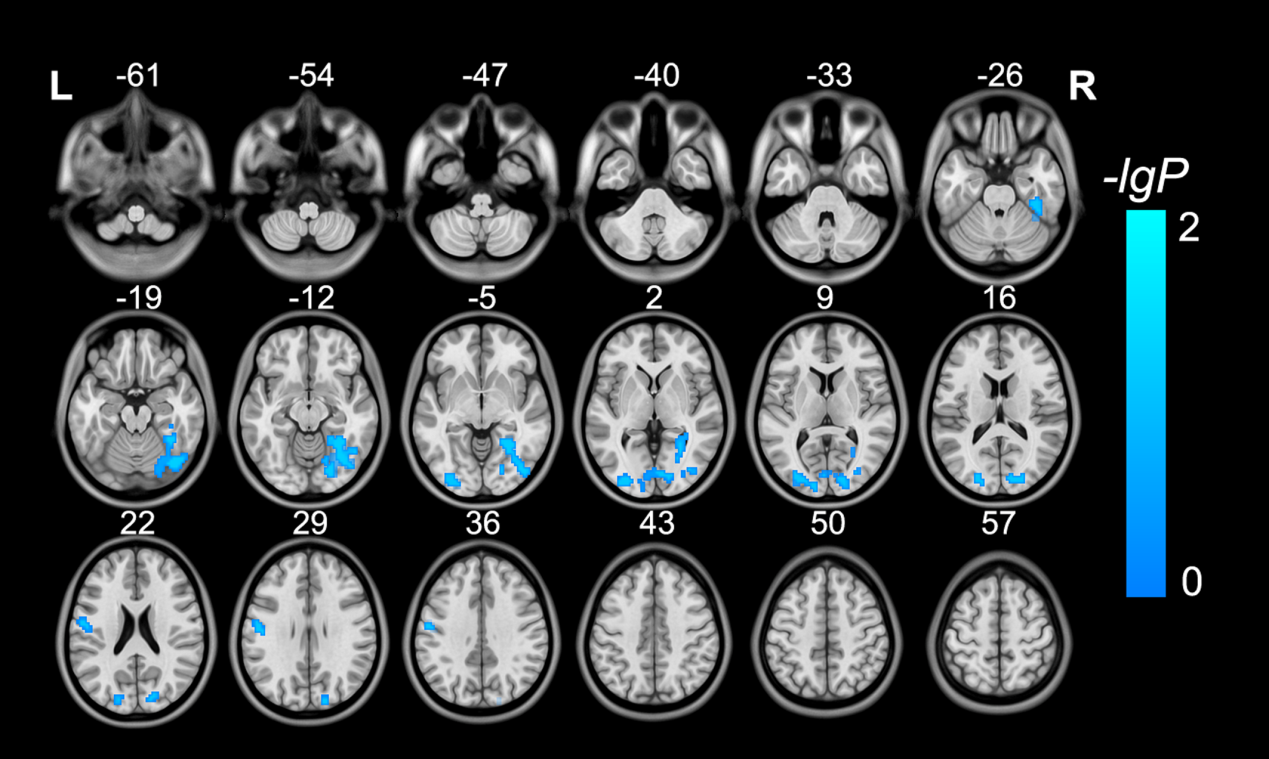


**Figure S1.** Brain regions exhibiting significant differences (*P* < 0.05, FWE corrected) in rs-gFCD between patients with MDD and healthy subjects in the nonparametric statistics. The coloured bar represents the *–lgP* value. rs-gFCD, resting-state global functional connectivity density; MDD, major depressive disorder; FWE, family-wise error.
