# Supplementary material for: Different spatial patterns of brain atrophy and global functional connectivity impairments in major depressive disorder
Source: Brain Imaging Behav. 2016 Oct 20;11(6):1678–89. doi: 10.1007/s11682-016-9645-z (PMC5707231; doi:10.1007/s11682-016-9645-z)
Supplement: Supplementary file 3 — (DOCX 799 kb) [file 11682_2016_9645_MOESM3_ESM.docx]

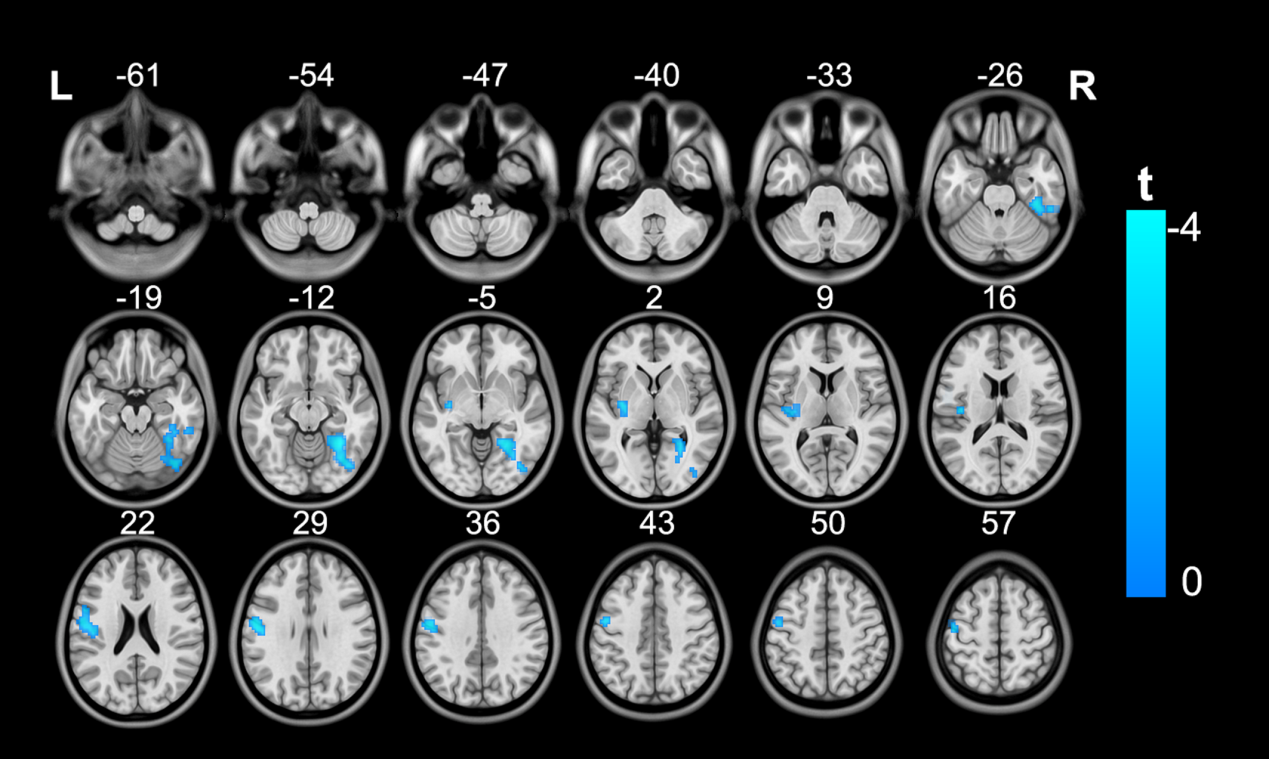


**Figure S3.** Brain regions with significant decreases in rs-gFCD at the correlation threshold of r > 0.4 between patients with MDD and healthy subjects. Colour bar represents the *t* value. Abbreviations: rs-gFCD = resting-state global functional connectivity density; MDD = major depressive disorder.
